# Efficient and Practical Correlation Filter Tracking

**DOI:** 10.3390/s21030790

**Published:** 2021-01-25

**Authors:** Chengfei Zhu, Shan Jiang, Shuxiao Li, Xiaosong Lan

**Affiliations:** 1 Institute of Automation, Chinese Academy of Sciences, Beijing 100190, China; chengfei.zhu@ia.ac.cn (C.Z.); jiangshan2017@ia.ac.cn (S.J.); shuxiao.li@ia.ac.cn (S.L.); 2 School of Artificial Intelligence, University of Chinese Academy of Sciences, Beijing 100049, China

**Keywords:** visual tracking, correlation filter, model update, long-term tracking

## Abstract

Visual tracking is a basic task in many applications. However, the heavy computation and low speed of many recent trackers limit their applications in some computing power restricted scenarios. On the other hand, the simple update scheme of most correlation filter-based trackers restricts their robustness during target deformation and occlusion. In this paper, we explore the update scheme of correlation filter-based trackers and propose an efficient and adaptive training sample update scheme. The training sample extracted in each frame is updated to the training set according to its distance between existing samples measured with a difference hashing algorithm or discarded according to tracking result reliability. In addition, we expand our new tracker to long-term tracking. On the basis of the proposed model updating mechanism, we propose a new tracking state discrimination mechanism to accurately judge tracking failure, and resume tracking after the target is recovered. Experiments on OTB-2015, Temple Color 128 and UAV123 (including UAV20L) demonstrate that our tracker performs favorably against state-of-the-art trackers with light computation and runs over 100 fps on desktop computer with Intel i7-8700 CPU(3.2 GHz).

## 1. Introduction

Visual object tracking is a branch of video analysis as the foundation of high-level image understanding. The main task of visual tracking is to continuously estimate the state (position and size) of the object in the subsequent frames given the initial state in the first frame. Many challenging factors, such as illumination variation, occlusion, fast motion, deformation and distractor may lead to tracking failure. In recent years, visual tracking algorithms have made considerable progress in terms of tracking accuracy and robustness with the efforts of researchers, and constantly refresh the upper limit of performance in various benchmarks and challenges. However, in many applications, such as UAV (unmanned aerial vehicle) tracking and edge computing, performance of the tracker is not the most important factor limiting its practicability, efficiency is also very important in practice.

In recent years, discriminative correlation filters (DCF) [1]-based visual tracking algorithms have been attracting the attention of researchers for its outstanding performance and speed. Correlation filter transforms spatial correlation operation into element-wise operation in frequency domain through fast Fourier transform to improve efficiency. However, traditional DCF trackers such as [2] are affected by boundary effects, while other improved DCF trackers [3,4] suffer from a large amount of calculations or unsatisfied model updating strategy, which cannot meet the requirements of target tracking when the computing resources are strictly restricted. In order to achieve balance between performance and efficiency of the tracker, we borrow the idea of mean shift tracking algorithms [5,6] to build a new color ratio feature (CR), and propose a DCF-based tracker embedded with CR features, namely CRCF tracker [7], which can achieve robust performance with real-time speed. Although our previous works have made great progress, nevertheless, a simple moving average update scheme of CRCF tracker cannot deal with the problem of occlusion and large appearance variation, and usually leads to model contamination and tracking drift.

In this paper, to address the aforementioned issue, we focus on the update scheme of CRCF tracker and design an efficient and adaptive training sample update scheme. On this basis, we propose a multi-training samples DCF-based tracker with outstanding performance and light computation trained on the training set with representative appearances of the target, namely CRCF_ATU. In addition, we expand CRCF_ATU to long-term tracking considering practical applications, on the basis of CRCF_ATU and its model updating mechanism, we propose a new tracking state discrimination mechanism to accurately judge tracking failure, and then we perform global redetection, and resume tracking after the target is recovered. Experiments on OTB-2015 [8], Temple Color 128 [9] and UAV123 [10] demonstrate that our CRCF_ATU tracker brings notable performance gain to CRCF tracker without reducing efficiency, and experimental results on UAV20L [10] show that the extended long-term tracker can effectively deal with the situation of long-term occlusion or target out-of-view, and successfully retrieve the target after the target reappears.

The main contributions of this paper are as follows: (1) We develop an efficient correlation filter-based tracker with outstanding performance, which is suitable for real-time applications. (2) We adaptively update training set and measure sample distance with difference hashing algorithm (DHA) [11]. (3) We train a multi-training sample DCF on the training set which exhibits better generalization ability. (4) We propose a DCF-based long-term tracking framework, which can effectively deal with the problem of long-term occlusion or out-of-view.

We note that a conference version of this paper appeared in reference [12]. The work in this paper is distinguished from prior work. Our initial conference paper just focused on the model update scheme of the tracker in short-term tracking, and did not pay attention to the redetection and tracking recovery of lost targets in long-term tracking. In this manuscript, we bring CRCF_ATU into the field of long-term tracking and propose a more effective tracking state discrimination mechanism based on the model update scheme of CRCF_ATU. When the target is judged to be lost, we try to retrieve the target using the global target redetector trained on the sample set, and then resume tracking after the target is redetected.

The rest of this paper is organized as follows: Section 2 introduces related works, Section 3 details the proposed method. Experiments to verify the proposed algorithm are illustrated in Section 4. Finally, Section 5 gives the main conclusions.

## 2. Related Works

### 2.1. Correlation Filter-Based Trackers

Since Bolme et al. [1] firstly introduced correlation filtering into visual tracking and proposed MOSSE tracker (minimum output of sum of squared error), DCF-based trackers have attracted the great attention of researchers. On the basis of [1], Henriques et al. proposed the circulant structure of tracking-by-detection with kernels (CSK) [13] to improve the classification performance. Then, they extended grayscale feature to multi-channel HOG feature and proposed the kernelized correlation filter (KCF) [2] to further improve the performance of tracking. Henceforth, DCF-based trackers have become one of the main streams in the field of visual tracking.

To solve the problem of scale estimation of correlation filtering with single template, the discriminant scale space tracker (DSST) [14] was proposed, which learned a one-dimensional scale filter on the multi-scale pyramid to detect scale change. Following this, fDSST [15] further improved the speed and performance of scale estimation by sub-grid interpolation of correlation scores and reduced the feature dimensionality using principal component analysis. Li et al. [16] transformed the sample into log polar space and estimated the scale and rotation of the target by phase correlation.

In terms of feature representation, Danelljan et al. [17] brought color names (CN) feature into correlation tracking, and improved the performance of the tracker by using color information of target and background. Bertinetto et al. [18] proposed a staple tracker, which combines the shape invariant color histogram response with the correlation filter response by weighted fusion, thus it could maintain real-time tracking performance and greatly improve tracking effect. With the rapid development of deep learning, convolutional neural network is also used for feature extraction of correlation tracking algorithms [19,20,21]; the performance of tracking algorithms is further improved through the semantic information contained in the convolution feature, but high computational complexity brought by feature extraction also reduces the efficiency of the algorithms.

For the boundary effects incurred by periodic assumption, spatially regularized discriminative correlation filter (SRDCF) [3] penalizes the regions far away from the target, which enhances the discrimination of the filter and expands the search range of the tracker. Based on SRCDF, C-COT [19] enables the efficient integration of multi-resolution deep feature maps with a continuous form of correlation filtering. Background-aware correlation filters (BACF) [22] enable the filter to learn more real negative samples through clipping operation. Dai et al. [23] used adaptive spatial regularization to further improve the performance of the filter.

To prevent model contamination and retain historical information, conventional DCF trackers [1,15] updated the numerator and denominator of the filter by moving average with a fixed update rate. However, the fixed update rate cannot deal with some special situations such as the rapid deformation and occlusion of the target. Other trackers [24,25] adopt a high-confidence update strategy to stop the model update or reduce the update rate when the tracking result is not reliable. C-COT [19] explicitly maintains a large training set with decaying weights and [26] proposed an adaptive training set decontamination strategy by jointly learning the correlation filter and sample weights. ECO tracker [27] maintains the training set with generative sample space model and trains the correlation filter at sparser intervals. Li et al. [28] maintained a FIFO sample queue to retain the historical typical state of target, and trained the filter in the form of multi-sample training set to expand the historical memory of the tracker.

### 2.2. Long-Term Tracking

Short-term tracking usually assumes that the target is always in the field of view, and does not care for target reposition after target tracking failure. Meanwhile, long-term tracking needs to deal with the situation that the target is completely blocked or out-of-view; it is required to accurately detect tracking failure or target disappearance, and reposition the target when the target appears again and resume tracking. Ma et al. [29] proposed a long-term correlation tracking algorithm, which uses the peak value of response map as the confidence, trains the detector based on random ferns classifier at high confidence level, and uses the detector to redetect the target when tracking confidence is low. Hong et al. [30] proposed the MUSTer algorithm, which has the ability to recover from tracking failure. Based on the memory model, a short-term memory module based on correlation filter tracking and a long-term memory module based on SIFT [31] local features are maintained, the central control module synthesizes the output of short-term memory module and long-term memory module to determine the final tracking output. Liu et al. [32] added an error correction mechanism in correlation filtering tracking, and used edge boxes [33] and SVM to generate instance region candidates and redetect them when tracking confidence is low. Wang et al. [24] combined correlation filter response and color response to judge tracking reliability; when tracking state is unreliable, the target candidate position is generated by particle filter. Sparse reconstruction is used to measure the quality of candidate position and high-quality candidate position is selected for redetection. Fan and Ling [34] proposed a parallel tracking and verification framework (PATV), which uses correlation filtering to track, and uses a more discriminative siamese network to verify the tracking results within certain frame intervals. LUKEZIC et al. [35] and BHAT et al. [36] used a spatial regularized correlation filter for tracking, and extended the search range to whole image by adding zeros to the filter in spatial domains. Ref [37] systematically analyzed the problems in long-term tracking, and gave a more scientific classification of the long-term tracking characteristics of tracking algorithms. They collected and annotated the LTB50 data set, and introduced evaluation indicators such as tracking accuracy, tracking recall rate and tracking F-score to evaluate the performance of long-term tracking algorithms more systematically.

## 3. The Proposed Method

In this section, we first give a brief introduction of our previous CRCF tracker [7]. Then, we present the overall process of our CRCF_ATU tracker with an adaptive training sample update scheme and describe some details. Finally, we extend the CRCF_ATU to long-term tracking, and discuss the mechanism of tracking state discrimination and the strategy of global redetection.

### 3.1. CRCF Tracker

In order to make better use of target and background color information to improve the performance of tracker, we borrow the weight map idea of mean shift algorithm to propose CR features [7], and integrate them into the correlation tracking framework to obtain the CRCF tracker, which improves the tracking speed and performance.

CR feature is obtained by comparing the target model with the target background model. Both the target model and the target background model are color histograms with *m* = 16 × 16 × 16 bins. Note that the target center is $x_{0}$, the target bandwidth is *h* and the target background bandwidth is *s* × *h* (*s* > 1). Then, the target model q[u] and target background model $p_{s}\left[u\right]$ can be calculated by the following formulas:

(1) $$\begin{array}{c} q\left[u\right]=C\sum_{i=1}^{n}k(∥\frac{x_{i}-x_{0}}{h}{∥}^{2})\delta \left[b\left(x_{i}\right)-u\right],u=1,2,...,m\\ p_{s}\left[u\right]=C_{s}\sum_{i=1}^{n_{s}}k(∥\frac{x_{i}-x_{0}}{s\cdot h}{∥}^{2})\delta \left[b\left(x_{i}\right)-u\right],u=1,2,...,m \end{array}$$

where *C* and $C_{s}$ are the normalization factors, k(·) is kernel function, b(x) is the histogram subscript corresponding to the color of pixel x, and δ[·] is impulse function, *n* and $n_{s}$ are the number of pixels in target area and target background area respectively, m=4096 is the number of subscripts in the histogram. For the histogram color index *u*, the weight can be calculated as follows:

(2) $$w\left[u\right]=\sqrt{q\left[u\right]/p_{s}\left[u\right]}$$

It has been proven in [6] that w[u] is bounded by [0,*s*]. Thus, we can limit the value range of w[u] to [0,1] by dividing *s* to get the CR features map, which can be described as $CR_{s}\left[u\right]=w\left[u\right]/s$. The target model and target background model are updated in each frame with learning rate $\eta_{CR}$ to adapt to target appearance variation.

**Figure 1:**
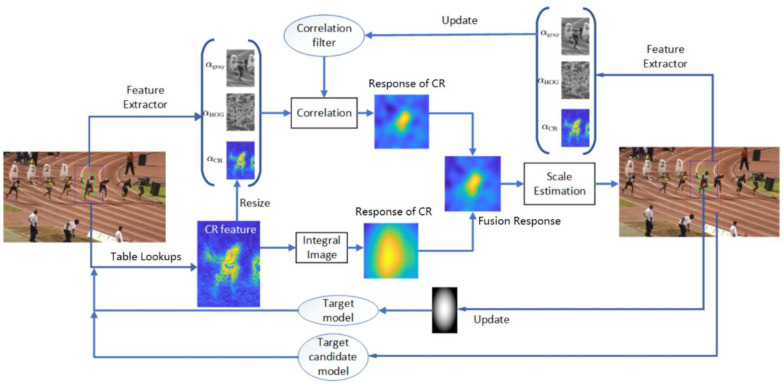
The overall sketch of CRCF tracker.

The algorithm flow of CRCF tracker is shown in Figure 1. In each frame, grayscale, 13 channels HOG [38] and color ratio (CR) features are extracted at the previous tracking result. The CR feature is simultaneously concatenated with HOG and grayscale feature to form 15 channel discriminative features for correlation filter and used to compute dense color based response. It has been proven in [18] that color-based response is invariant to deformation, which is complementary to template-based correlation filter response. Besides, compared to the correlation filter model, which adapts cosine window and limits the tracker search region, color-based model has a larger search region, enabling the tracker to handle fast motion to some extent. As shown in Formula (3), CRCF tracker integrates the color dense response $R_{CR}$ and the correlation filter response $R_{CF}$ to estimate the target displacement, where γ is the fusion coefficient.

(3) $$R=\left(1-\gamma \right)R_{CF}+\gamma R_{CR}$$

The detailed description of the algorithm can be found in [7].

### 3.2. CRCF_ATU Tracker

**Figure 2:**
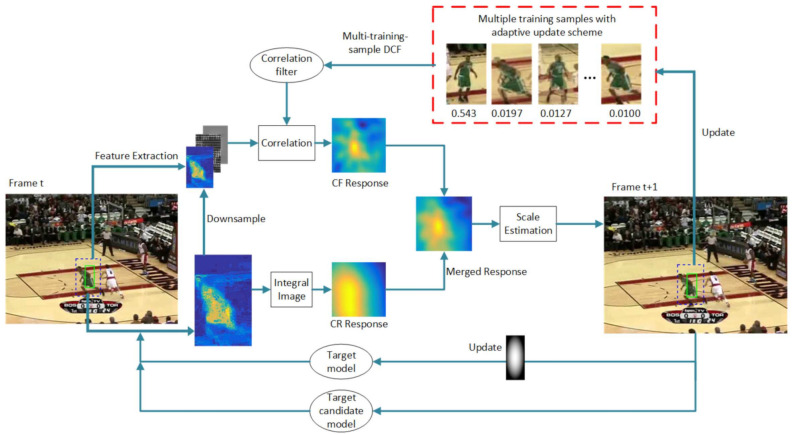
The overall sketch of the proposed CRCF_ATU tracker.

Aiming at the problem of model drift in correlation tracking model updating, CRCF_ATU is proposed on the basis of the CRCF [7] and the adaptive samples set the updating mechanism based on the difference hashing algorithm (DHA) [11]. Unlike our previous work which trains and updates correlation filter in each frame, we adaptively maintain a training samples set and train the correlation filter on it. The training sample is extracted from the tracking result in each frame and is added or discarded to the training set according to our new adaptive update scheme. The overall sketch of the proposed CRCF_ATU tracker is shown in Figure 2.

For the training sample set of size *N*, we follow [27] to employ the generative Gaussian model to maintain and update a compact training set. Each training sample is denoted as $x_{k}$ and its corresponding weight is $\alpha_{k}$. The training sample weights sum up to 1. The update rate of the sample set is denoted as η. Each time that a new sample is updated to the training set, the weight of the new sample is η and the weights of the original samples are multiplied by (1−η). The updating rules of the sample set are as follows:

1. If the number of samples in the training set is less than *N*, the new sample is added into the training set.
2. If the number of training samples exceeds *N* and the minimum weight is below the forgetting threshold, the sample with minimum weight is replaced by the new sample.
3. If the number of training samples exceeds *N* and there is not any sample’s weight below the forgetting threshold, the closest two samples are merged into one sample.

The training set stores representative appearances of the target and the correlation filter is trained on the training set at sparser intervals. This update strategy enables the correlation filter with better generalization ability and reduces the computational burden at the same time.

However, in [27], the distance between samples is measured with Euclidean distance and requires one to compute inner product between samples. In addition, background region on the sample patch is also taken into account, which is not reasonable. On the basis of [27], we adopt a difference hashing algorithm (DHA) [11] to measure the distance between samples and only the target region in the sample patch is taken into account. DHA calculates the hashing matrix for each image, and measures the similarity of two images by the Hamming distance between their hashing matrixes. Each sample patch is converted to grayscale and resized to 8×9 and denoted as $B\in R^{8\times 9}$, and the hashing matrix $H\in R^{8\times 8}$ can be computed as:

(4) $$h_{i,j}=\left\{\begin{array}{c} 1,\;if\;b_{i,j}>b_{i,j+1}\\ 0,\;\;otherwise \end{array}\right.$$

where $b_{i,j}$ and $h_{i,j}$ denote the element on the *i*-th row and *j*-th column of *B* and *H*. Each image patch can be represented with this hashing matrix and the distance between two image patches can be measured with the hamming distance between their corresponding hashing matrix $H^{c}$ and $H^{l}$,

(5) $$d_{lc}=\sum_{i=1}^{8}\sum_{j=1}^{8}\left(h_{ij}^{c}⊕h_{ij}^{l}\right)$$

where ⊕ is XOR operator, the bitwise operation enables distance measurement to be efficient. The update mechanism of training samples is improved based on the DHA measure. For each sample, the target region is clipped to calculate its hashing matrix. The speed and accuracy of calculating the distance between samples could be improved by extra space storage.

Referring to the scheme of online maintenance of training sample set in [3,19,27], we maintain a multi-sample training set to save the typical appearances of the target, and incorporate those training samples into traditional DCF formulation, which is to minimize the following objective function,

(6) $$E\left(h\right)=\sum_{k=1}^{N}\alpha_{k}∥\sum_{d=1}^{D}h_{d}*x_{kd}{-y∥}^{2}+\lambda {∥h∥}^{2}$$

where ∗ denotes cross-correlation operator, $h_{d}$ and $x_{kd}$ are the *d*-th channel of the correlation filter *h* and the training sample $x_{k}$ respectively. *y* is the gaussian label and $\alpha_{k}$ is the weight to sample $x_{k}$. *N* is the number of samples, *D* is the number of feature channels, and λ is the regularization factor. This minimizer has a closed-form solution in Fourier domain as,

(7) $${\hat{h}}_{d}=\frac{\sum_{k=1}^{N}\alpha_{k}{\hat{x}}_{kd}^{*}⊙\hat{y}}{\sum_{k=1}^{N}\alpha_{k}\sum_{d=1}^{D}{\hat{x}}_{kd}^{*}⊙{\hat{x}}_{kd}+\lambda }$$

where ^ denotes the discrete Fourier transform (DFT), ${\hat{x}}_{kd}^{*}$ is the complex-conjugate of ${\hat{x}}_{kd}$ and ⊙ denotes element-wise multiplication. This formulation incorporates multiple training samples into DCF formulation without increasing time complexity.

To achieve robust target tracking, it is necessary to judge the reliability of tracking results, and stop updating the model when the tracking results are unreliable. The reliability of results in correlation, tracking can be measured by the peak value and fluctuation degree of the response map. For CRCF_ATU, the fluctuation degree of response map can be measured by APCE (average peak correlation energy).

(8) $$APCE\left(R\right)=\left(R_{max}-R_{min}\right)/\sum_{i,j}\frac{R_{i,j}-R_{min}}{mn}$$

where $R_{max}$ and $R_{min}$ are the maximum and minimum values of the response map respectively, $R_{i,j}$ is the element of the *i*-th row and *j*-th column on the response map, *m* and *n* are the width and height of the response map respectively. It is found that the product of peak value of response map and APCE can better reflect the reliability of response map, which can be calculated as τ(R)=APCE(R)·max(R). We notice that the CRCF_ATU depends on the complementary of correlation filter response, color response and the merged response to determine the target position; when one is unreliable, another one can be used to track the target. Therefore, the judgment criteria is as follows: tracking result is considered to be unreliable only when the confidence of correlation filter response $\tau (R_{CF})$, color response $\tau (R_{CR})$ and fusion response τ(R) are all significantly lower than the historical average:

(9) $$\frac{\tau (R_{CF})}{\tilde{\tau }\left(R_{CF}\right)}<T_{CF}\;\frac{\tau (R_{CR})}{\tilde{\tau }\left(R_{CR}\right)}<T_{CR}\;\frac{\tau (R)}{\tilde{\tau }\left(R\right)}<T_{R}$$

where $T_{CF}$, $T_{CR}$ and $T_{R}$ are the ratio thresholds and $\tilde{\tau }\left(R\right)$ is the historical average of the reliability of response *R*.

### 3.3. Expanding to Long-Term Tracking

**Figure 3:**
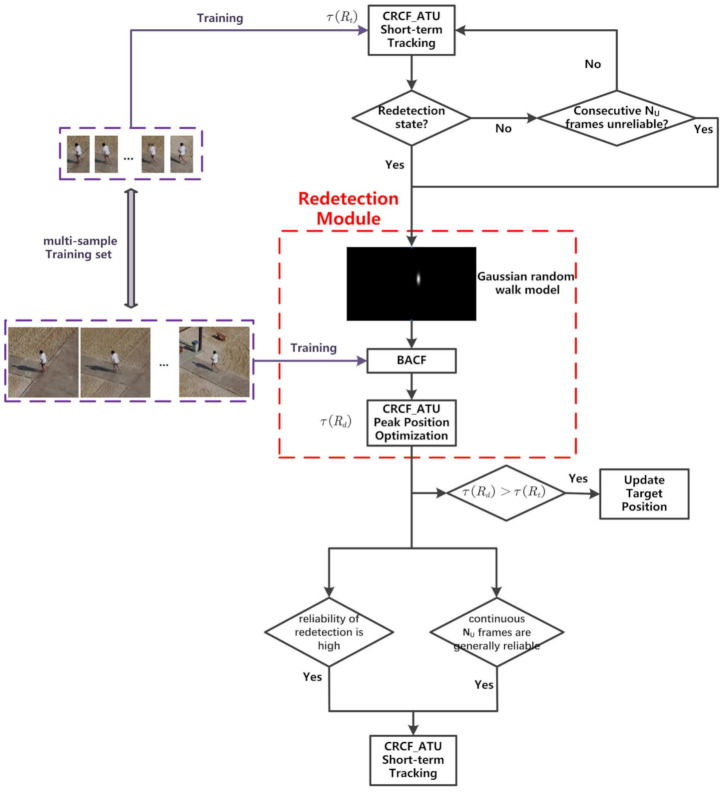
The overall sketch of the proposed CRCF_LCT tracker.

CRCF_ATU can deal with the situation of fast deformation or partial occlusion of the target; when the target is always in the field of view, it can achieve fast and robust target tracking. However, when the target is fully occluded for a long time or out of view and then returns to the field of view again, CRCF_ATU cannot successfully continue tracking. In order to address the above issues, we extend CRCF_ATU tracker to propose a long-term tracker named CRCF_LCT. Figure 3 shows the flowchart of the proposed CRCF_LCT tracker.

One of the main problems in long-term tracking is how to judge tracking failure, and when to enable and stop global redetection. For our CRCF_LCT tracker, according to the criterion of the reliability of CRCF_ATU tracking results which is shown in Formula (9), tracking failure is determined only when continuous $N_{U}$ frames are determined to be unreliable. Then, the long-term tracking process switches to the redetection state.

In the Redetection Module of Figure 3, the last reliable tracking position is taken as the center to continuously expand the search range. The above a priori information is used to determine the extent of global redetection by Gaussian random walk model $N(x_{t};x_{c},\Sigma_{t})$, where $x_{t}$ is the last reliable tracking position, $\Sigma_{t}=diag\left(\delta_{xt}^{2},\delta_{yt}^{2}\right)$ is the covariance matrix of random walk model. As frames increase in global redetection state, $\left[\delta_{xt},\delta_{yt}\right]=\left[w,h\right]\alpha_{s}^{\Delta t}$, where $\alpha_{s}$ is the scale factor and Δt is the number of frames. Then we employ BACF [22] detector to globally redetect the target in the image block with width and height $\left[\delta_{xt},\delta_{yt}\right]$.

BACF (background aware correlation filtering algorithm) [22] is an improved correlation filtering algorithm to solve the edge effect caused by cyclic shift hypothesis. BACF uses the binary mask matrix *P* to cut the central target area of the filter, so that the filter can learn more real negative samples in the training process, which enhances the discrimination of the filter. Clipping operation can not only alleviate the edge effect, but also expand the search range of the tracker, and enhance the tracker’s ability to deal with rapid movement of the target.

Let *K* donate the number of channels, when detecting the target in the next frame, the trained filter $\hat{g}$ and the next frame feature *Z* are used to obtain the response map as follows:

(10) $$f\left(z\right)=F^{-1}\sum_{k=1}^{K}\left({\hat{g}}_{k}^{*}⊙{\hat{z}}_{k}\right)$$

**Figure 4:**
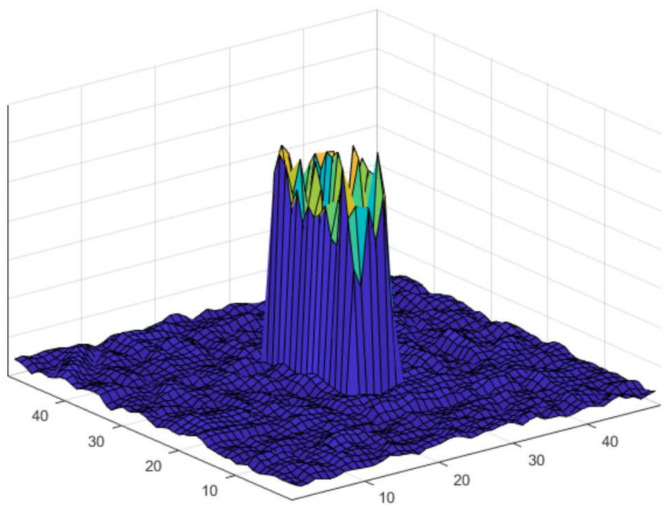
Visualization of the filter coefficients of BACF.

The coefficients of spatial filter trained by BACF algorithm are shown in Figure 4. We can see that the coefficients outside the target area are 0, therefore, the search range of the filter can be extended by spatial zero padding to achieve global redetection.

While CRCF_ATU tracker maintains the training sample set, each sample retains a larger copy image block to train the BACF detector. When the Redetection Module is activated, the feature is extracted from large image blocks, and weighted sum of corresponding samples in the sample set are used to train the BACF filter. Finally, the target is detected globally by the BACF filter, which is expanded to the image size by zero padding. At the peak position of global detection, CRCF_ATU tracker is used again to optimize the position. If the response reliability of CRCF_ATU tracker $\tau (R_{d})$ at the optimized position is greater than the response reliability of the current frame tracker $\tau (R_{t})$, target position is updated to the optimized peak position. In the global redetection state, if the reliability of the redetect result is high or the detection results of continuous $N_{U}$ frames are judged to be generally reliable (i.e., not meeting Formula (9)), the global redetection is stopped and the state is switched back to CRCF_ATU short-term tracking mode. The criterion of the high reliability of redetection result is to meet Formula (11).

(11) $$\frac{\tau (R_{CF})}{\tilde{\tau }\left(R_{CF}\right)}\geq T_{CF}\;\frac{\tau (R_{CR})}{\tilde{\tau }\left(R_{CR}\right)}\geq T_{CR}\;\frac{\tau (R)}{\tilde{\tau }\left(R\right)}\geq T_{R}$$

The meanings of the symbols in Formula (11) are the same as those in Formula (9).

## 4. Experiments

### 4.1. Experimental Setup

For the CRCF_ATU tracker, we conduct extensive experiments on three challenging benchmarks, including OTB-2015 [8], Temple Color 128 [9] and UAV123 [10]. For CRCF_LCT tracker, we evaluate its long-term tracking capability on dataset UAV20L [10]. All experiments are conducted on a desktop computer with Intel i7-8700 CPU (3.2 GHz) and 16 GB memory. Following [8], we use precision plot and success plot to evaluate trackers. Precision plot measures the ratio of the frames of which center location error are under a series of thresholds. Success plot measures the ratio of the frames of which overlap between the ground truth bounding boxes and the ones generated by the trackers are over a series of thresholds. In our experiment, precision plot and the precision at the threshold of 20 pixels (denoted as P20) are used to evaluate the precision of the trackers, success plot and the area under curve (AUC) of the success plot are used to evaluate the accuracy of the trackers.

### 4.2. Implementation Details

Our trackers are implemented with Matlab and the parameters are kept fixed for all sequences. $T_{CF}$, $T_{CR}$ and $T_{R}$ in Formulas (9) and (11) are set to 0.6, 0.7 and 0.6. The CR model learning rate $\eta_{CR}$ is set to 0.04. The training set size *N* is set to 31 and learning rate η is set to 0.01. The response merge factor γ is set to 0.3. The correlation filter is trained on the training set every 5 frames. The HOG feature is of 13 channels and is implemented with modified Piotr’s toolbox [39]. For scale estimation, we use fDSST [15]. The parameter setting of the BACF for redetection is the same as [22]. Specifically, the standard deviation of Gaussian objective function is set as $\delta =\sqrt{wh}/16$, where *w* and *h* are the width and height of the target. Regularization factor λ is set to 0.001, the iterations of the alternating direction method of multipliers (ADMM) are set to 2, the penalty coefficient μ is set as 1, The penalty coefficient is updated iteratively as $\mu^{\left(i+1\right)}=\min\left(\mu_{max},\beta \mu^{\left(i\right)}\right)$, where β=10, $\mu_{max}=10^{3}$. The scale growth factor of Gaussian random walk model $\alpha_{s}$ is set to 1.05, and the cumulative number of frames $N_{U}$ is set to 5.

### 4.3. Comparative Evaluation of Update Mechanism

To validate the effectiveness of the update mechanism of our CRCF_ATU tracker, we compare the DHA-based samples update mechanism proposed in this paper and the update mechanism in Reference [27] on OTB-2015 [8]. Experimental results are summarized in Table 1. CRCF+GMM represents CRCF tracker that adopts the update scheme of [27]. From the results, we can observe that the adaptive training sample update scheme brings a gain of 2.42% in precision and 1.81% in AUC, which exhibits better performance than CRCF+GMM. CRCF+GMM runs at relatively low speed due to the heavy computation of sample distance measurement. Therefore, our CRCF_ATU tracker brings performance gain without reducing the high speed of CRCF, achieving a balance between performance and efficiency.

### 4.4. Performance Verification

#### 4.4.1. Results on OTB-2015

**Figure 5:**
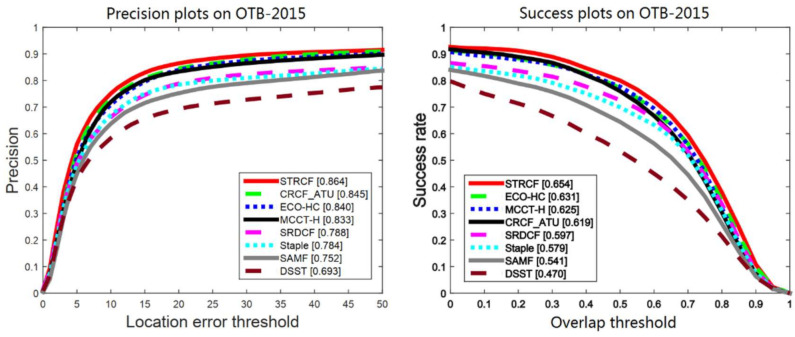
Comparison between CRCF_ATU tracker and existing trackers on OTB-2015.

**Figure 6:**
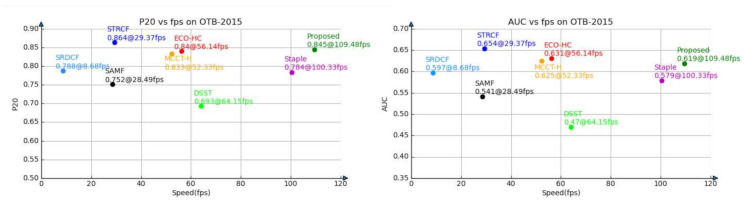
Performance efficiency plot on OTB-2015 of CRCF_ATU tracker and existing trackers.

OTB-2015 [8] is one of the most popular datasets in the visual tracking community, which consists of 100 challenging sequences with annotated challenging attributes. We compare our CRCF_ATU tracker with 7 recent correlation filter-based trackers, including STRCF [40], ECO-HC [27], MCCT-H [41], SRDCF [3], Staple [18], SAMF [42] and DSST [14]. The precision plot and success plot are shown in Figure 5. In addition, in order to better compare the performance and efficiency of various algorithms, we plot the tracker performance (P20 and AUC) versus speed (FPS) in Figure 6 and trackers on the top right corner exhibit better balance between performance and efficiency. Our proposed CRCF_ATU tracker exhibits a similar performance as MCCT-H with 2× fps. STRCF and ECO-HC with high performance solve the filter in an iterative manner and thus need more computation and are relatively slow. The experimental results show that the proposed CRCF_ATU tracker achieves the performance close to the best trackers at present, with relatively light computation.

#### 4.4.2. Results on Temple Color 128

**Figure 7:**
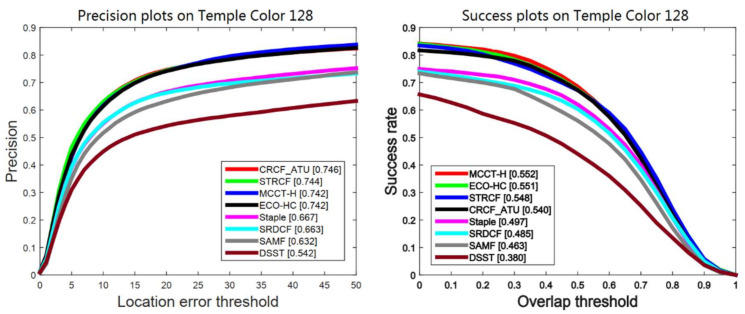
Comparison between our proposed CRCF_ATU tracker and existing trackers on Temple Color 128.

**Figure 8:**
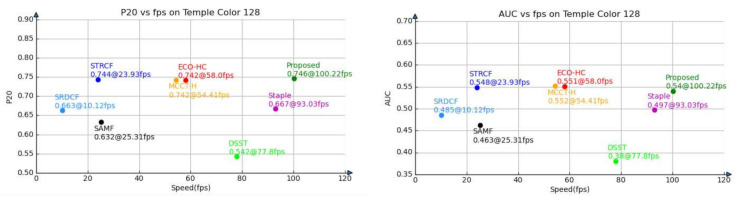
Performance-efficiency plot on Temple Color 128 of CRCF_ATU tracker and existing trackers.

Temple Color 128 [9] consists of 128 color video sequences. Our CRCF_ATU tracker is compared with seven competitive trackers, including STRCF [40], ECO-HC [27], MCCT-H [41], SRDCF [3], Staple [18], SAMF [42] and DSST [14]. Comparative results are shown in Figure 7 and Figure 8. Our CRCF_ATU tracker achieves close performance to STRCF, ECO-HC and MCCT-H. It should be noted that Temple Color 128 consists of color sequences, while OTB-2015 contains several grayscale sequences. Our CRCF_ATU tracker performs better on Temple Color 128 due to taking advantage of color information.

#### 4.4.3. Results on UAV123

**Figure 9:**
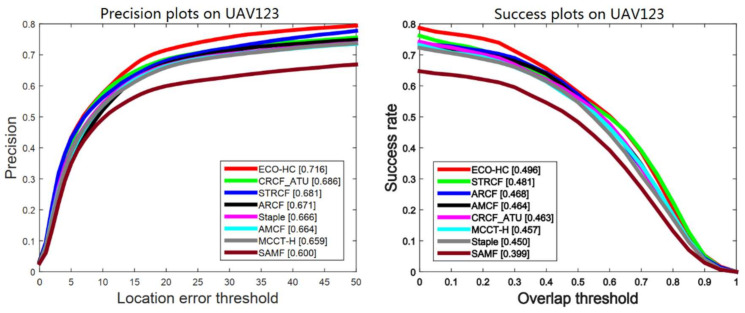
Comparison between CRCF_ATU tracker and existing trackers on UAV123.

**Figure 10:**
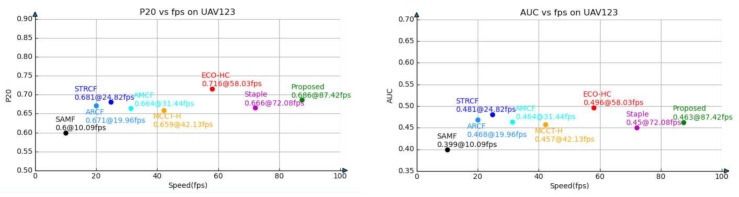
Performance efficiency plot on UAV123 of CRCF_ATU tracker and existing trackers.

UAV monitoring is one of the most important applications of visual tracking. UAV123 [10] consists of 123 real and simulated video sequences from aerial viewpoint, which is inherently different from general tracking datasets. Our CRCF_ATU tracker is compared with seven trackers, including STRCF [40], ECO-HC [27], ARCF [43], Staple [18], AMCF [28], MCCT-H [41] and SAMF [42]. As shown in Figure 9 and Figure 10, our CRCF_ATU tracker has a competitive performance to STRCF, ECO-HC and ARCF. This shows the effectiveness of our tracker under challenges of UAV scenarios (e.g., fast motion). However, MCCT-H with robust performance on other benchmarks has a relatively poor performance on UAV123, which demonstrates that the hypothesis of MCCT-H does not hold in UAV tracking.

#### 4.4.4. Long-Term Tracking Results on UAV20L

**Figure 11:**
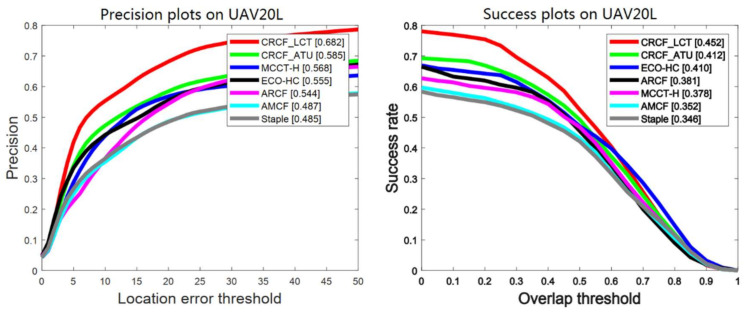
Comparison between CRCF_LCT tracker and existing trackers on UAV20L.

UAV20L is a subset of UAV123 [10], which contains 20 long video sequences with many cases of long-term occlusion and out of view, therefore, it can be used to evaluate the long-term tracking performance of the trackers. Our CRCF_LCT tracker is compared with six trackers, including MCCT-H [41], ECO-HC [27], ARCF [43], AMCF [28], Staple [18] and our short-term tracker CRCF_ATU. The experimental results are shown in Figure 11. Compared with other short-term tracking algorithms, the proposed algorithm can judge the tracking state accurately and retrieve the target successfully after long-term occlusion or out-of-view, thus greatly improving the performance in long-term video sequences.

#### 4.4.5. Qualitative Analysis

**Figure 12:**
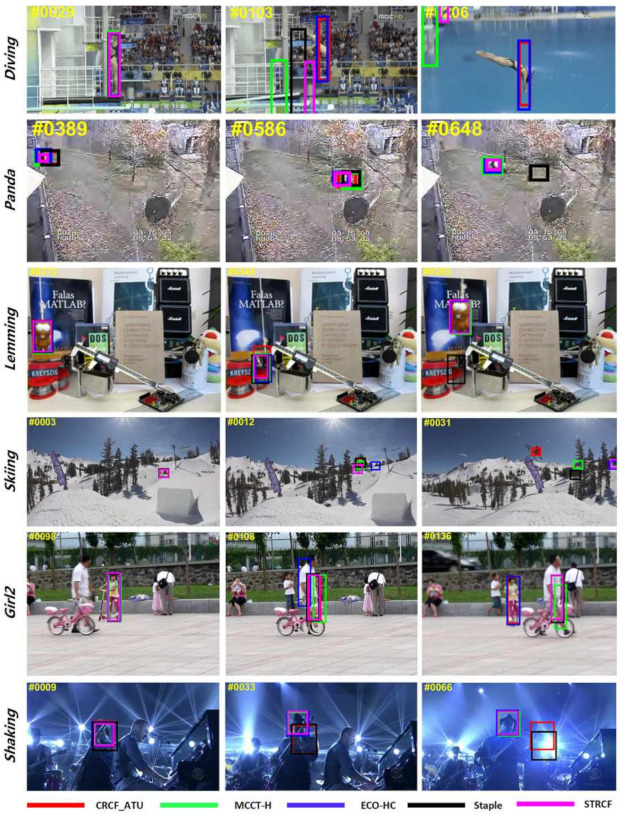
Tracking result visualization of our proposed CRCF_ATU tracker and comparative trackers on sequences Diving, Panda, Lemming, Skiing, Girl2, Shaking.

Figure 12 visualizes the tracking results on some challenging sequences from OTB-2015. From the sequences Diving, Skiing and Panda, we can see that the proposed CRCF_ATU tracker performs robustly against a large appearance variation for merging color-based response and adaptive update scheme. In sequences Girl2 and Lemming, the proposed tracker successfully recovers tracking after short-term occlusion. Sequence shaking shows a failure case in which illumination variation distracts the color histogram model and leads to tracker drift.

**Figure 13:**
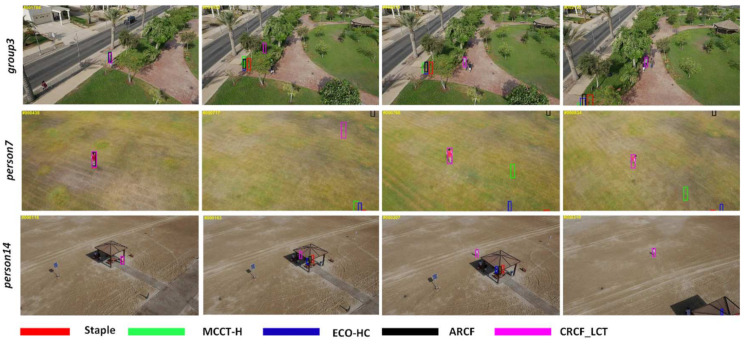
Tracking result visualization of our proposed CRCF_LCT tracker and comparative trackers on sequences group3, person7, person17.

Figure 13 shows the tracking results on three challenging sequences in UAV20L. Compared with other trackers, we can see that the proposed CRCF_LCT tracker can deal with complex situations, such as long-term occlusion and out of view. When the target returns to the field of view, the CRCF_LCT tracker can detect the target and resume tracking successfully.

## 5. Conclusions

In this paper, for the applications with restricted computing power, we propose an efficient training sample update scheme and adaptively maintain a training set using difference hashing algorithm. The filter with stronger discrimination and generalization ability is trained on the training set, which improves the performance of the CRCF_ATU tracker while maintaining high speed. For practical long-term tracking applications, a more accurate tracking state discrimination mechanism is proposed to judge the tracking state based on the update mechanism of our CRCF_ATU tracker. When the tracking failure is judged, the BACF with a wider search range is trained on the maintained sample set to conduct global redetection, and recover the short-term tracking after the target is found. Experimental results show that our tracker can achieve a close performance to state-of-the-art trackers, with relatively light computation and high speed. Moreover, the extended long-term tracker can accurately judge the tracking state and successfully retrieve the target when the target reappears. The balance between performance and efficiency enables our tracker to be practical in computation restricted applications.

## Figures and Tables

**Table 1:** Comparative results of model update scheme.

Method	P20	AUC	FPS
CRCF+GMM	0.825	0.608	90.32
CRCF_ATU	0.845	0.619	109.48
